# Supplementary material for: Rapid meniscus-guided printing of stable semi-solid-state liquid metal microgranular-particle for soft electronics
Source: Nat Commun. 2022 May 12;13:2643. doi: 10.1038/s41467-022-30427-z (PMC9098628; doi:10.1038/s41467-022-30427-z)
Supplement: Supplementary file 1 — Supplementary Information [file 41467_2022_30427_MOESM1_ESM.pdf]

Rapid meniscus-guided printing of stable semi-solid-state liquid metal microgranular-particle for soft electronics

Gun-Hee Lee[1,2,†], Ye Rim Lee[1,†], Hanul Kim[3], Do A Kwon[1], Hyeonji Kim[1], Congqi Yang[4], Siyoung Q. Choi[3,5], Seongjun Park[4,6], Jae-Woong Jeong[2,6,*], and Steve Park[1,6,*]

[1] Department of Materials Science and Engineering, Korea Advanced Institute of Science and Technology (KAIST), 291 Daehak-ro, Yuseong-gu, Daejeon 34141, Republic of Korea

[2] School of Electrical Engineering, Korea Advanced Institute of Science and Technology (KAIST), 291 Daehak-ro, Yuseong-gu, Daejeon 34141, Republic of Korea

[3] Department of Chemical and Biomolecular Engineering, Korea Advanced Institute of Science and Technology (KAIST), 291 Daehak-ro, Yuseong-gu, Daejeon 34141, Republic of Korea

[4] Department of Bio and Brain Engineering, Korea Advanced Institute of Science and Technology (KAIST), 291 Daehak-ro, Yuseong-gu, Daejeon 34141, Republic of Korea

[5] KAIST Institute for the NanoCentury, Korea Advanced Institute of Science and Technology (KAIST), Daejeon 34141, Republic of Korea

[6] KAIST Institute for Health Science and Technology, 291 Daehak-ro, Yuseong-gu, Daejeon 34141, Republic of Korea

[†] These authors contributed equally: Gun-Hee Lee, Ye Rim Lee.

Corresponding authors: J.-W.J (jjeong1@kaist.ac.kr), S.P. (stevepark@kaist.ac.kr)

	This work	Ref. 1-3	Ref. 4	Ref. 5-8	Ref. 9
**Strategy**	Meniscus-guided printing	Bulk	Hydrogen-doping	Mechanical activation	Chemical activation
**Physical state**	Semi-solid	Liquid	Particles embedded elastomer	Particles embedded elastomer, solid	Solid
**Initial conductivity**	○	○	○	X	X
**Additional process**	X	X	Annealing (120 °C, 3 h)	Applying mechanical stimuli	Exposing acidic fume
**Resolution**	High (~50 μm)	High	Low (>100 μm)	Low	Low
**Printability on various substrates**	○	X	X	X	X
**Multi-layering**	○	△	△	X	X
**Film thickness control**	○	X	X	X	X

Supplementary Table 1| Comparison between this work and reported liquid metal particle (LMP)-based fabrication strategy.

	Continuous phase (solvent)	Dispersed phase (liquid metal droplet)
	Aqueous acetic acid	EGaIn
Density, ρ (g/mL)	1	6.25
Viscosity, μ (mPa·s)	1.2	10
Interfacial tension, γ (mN/m)	430	

Supplementary Table 2| Liquid properties and interfacial tension.

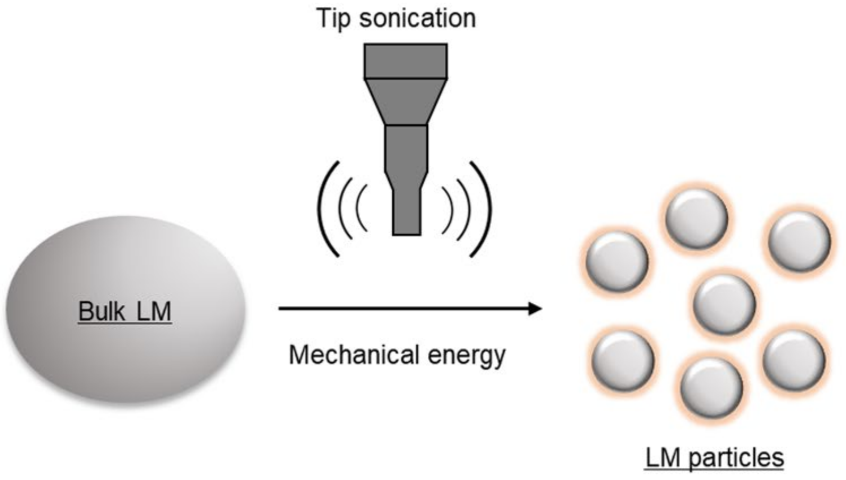

Supplementary Fig. 1| Schematic illustration of liquid metal particles (LMPs) preparation.

Bulk liquid metal (LM) is split into LMPs when the solution is tip sonicated. LMPs are then covered with a thin oxide layer that works as an insulator.

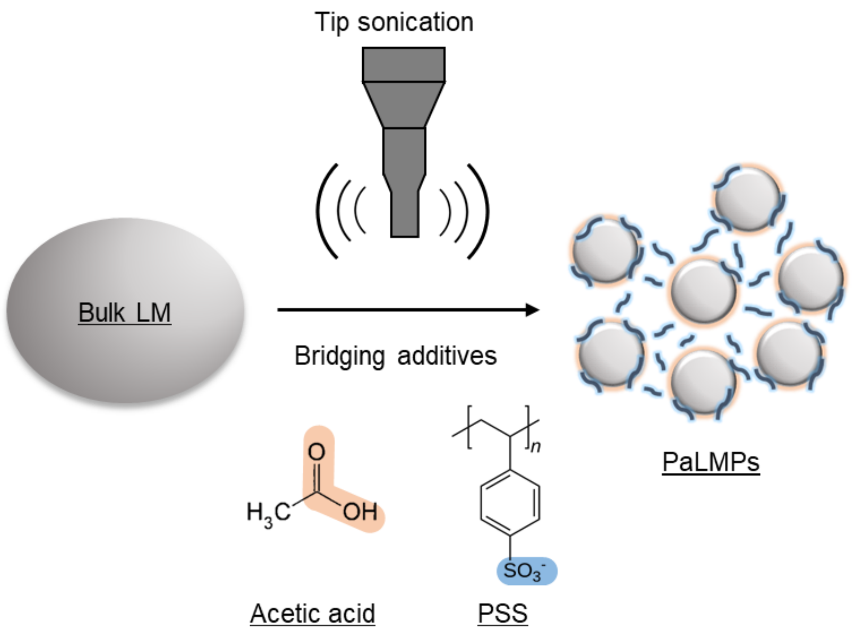

Supplementary Fig. 2| Schematic illustration of semi-solid-state liquid metal microgranular particles (PaLMPs) preparation.

PSS is added to wrap and bridge the LMPs during tip sonication. Acetic acid (AA) reduces the electrostatic repulsion between LMPs and PSS during thin-film deposition.

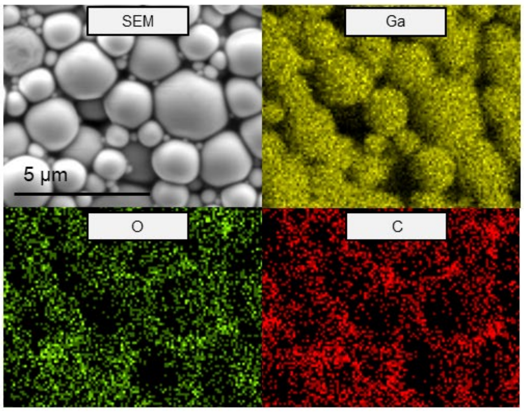

Supplementary Fig. 3| Energy dispersive X-ray spectroscopy (EDS) data of PaLMP.

Carbon, which is a main element of PSS, is dispersed on the surface of LM particles.

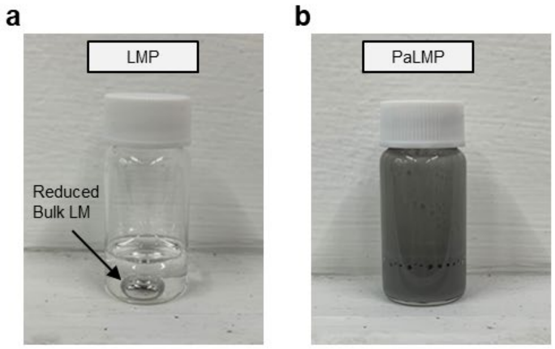

Supplementary Fig. 4| Chemical stability of ink according to the presence and absence of PSS. a, Photograph of reduced LMP into bulk LM when HCl was added to the solution without PSS. **b**, Photograph of the PaLMP ink after the addition of HCl.

To measure the stability of ink, 0.2 ml of HCl (37%) is added to 4 ml ink.

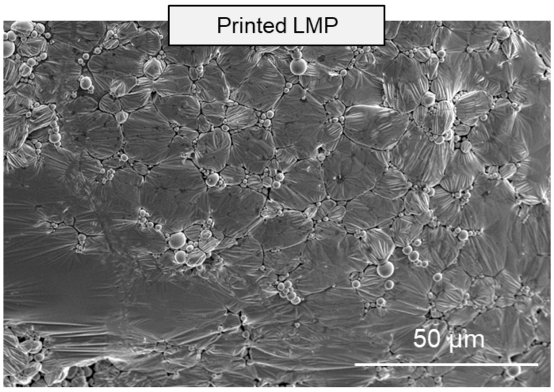

Supplementary Fig. 5| SEM image of clogged bare LMP.

Due to instability, bare LMPs ruptured and coalesced during printing, which results in the clogging of the nozzle.

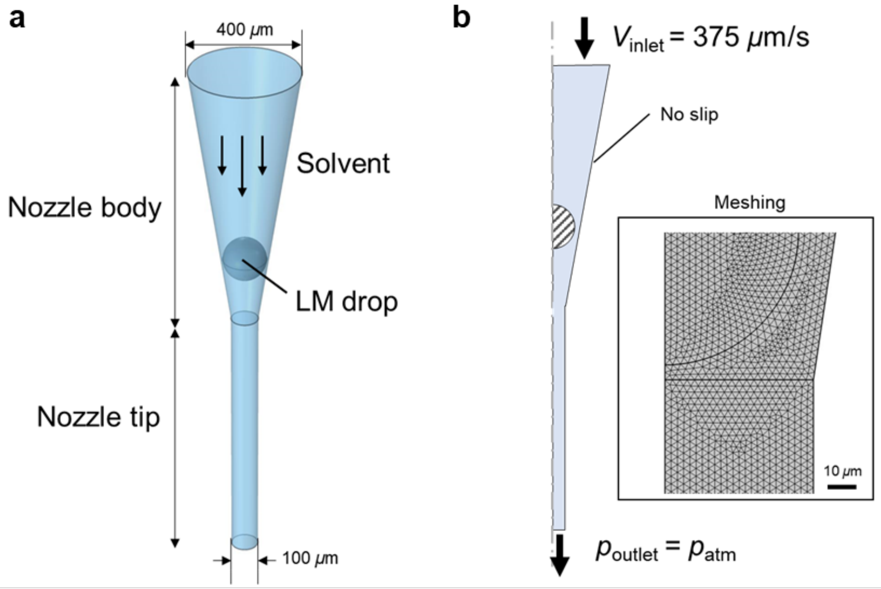

Supplementary Fig. 6| Schematics for numerical simulation conditions. **a**, Schematic of bottle neck containing the nozzle body and nozzle tip. **b**, The axi-symmetric 2D geometry and boundary flow conditions. The inset is the meshing result with the 'finer' option.

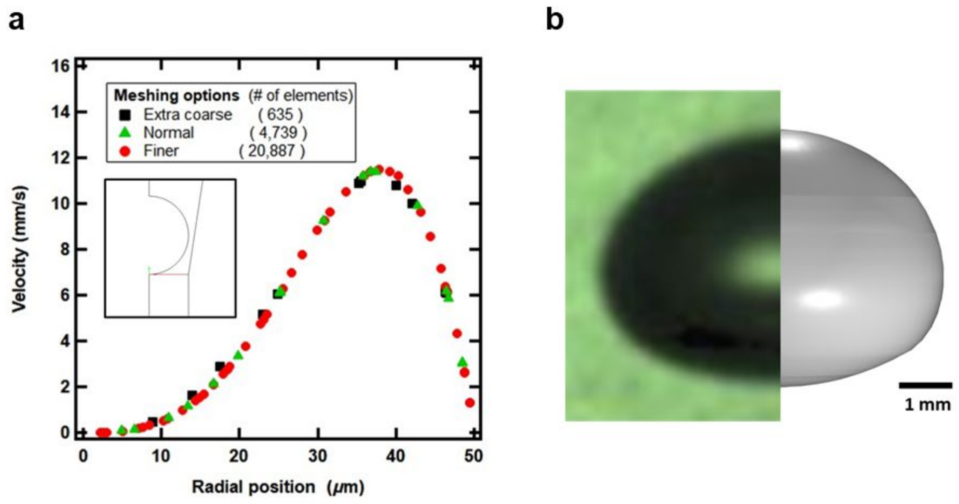

Supplementary Fig. 7| Validation of numerical simulation method. a, Mesh test for the fluid flow without liquid/liquid interface. (Inset) The red solid line is the sampling line of velocity profile for the mesh test. **b**, Experimental data (left) and numerical simulation result (right) of air bubble rising in aqueous glycerol solution.

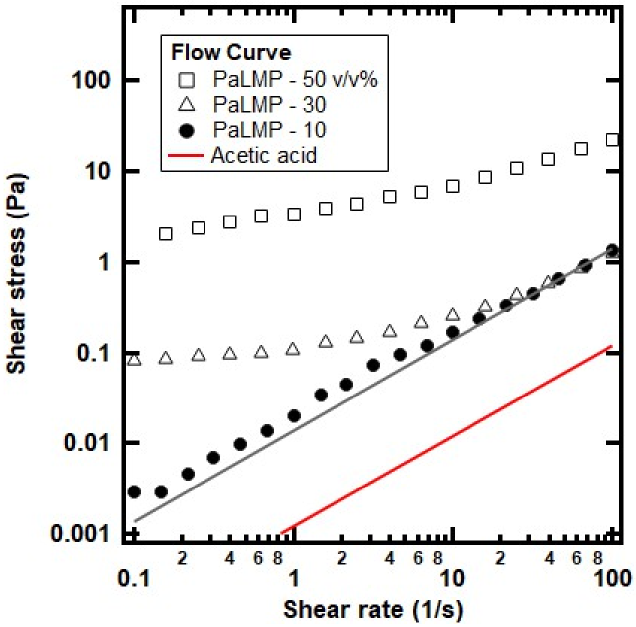

Supplementary Fig. 8| Rheological flow curve of PaLMP inks. Filled circles indicate flow curve of 10 v/v% PaLMP and gray line is its linear fit. Open data points are PaLMP with higher LM volume fractions (30 and 50 v/v %). The red line is the flow curve of acetic acid solution.

Rheological properties of PaLMPs were controlled by varying LM volume fraction. Shear rate-stress flow curve is measured with the strain stress control rheometer (MCR 302, Anton Paar). Shear rate range is from 0.1 to 102 1/s with logarithmic ramp up condition and the test time between each point was set as 60s to reach steady state. The torque limit was 0.1 μN·m which is sufficiently lower than the measuring range. Parallel plate (PP25) is used. For 10 v/v% sample, cylinder type cell (DG26.7/T200/SS) is used for the sufficient torque.

In **Supplementary Fig. 8**, the PaLMP sample with 10 v/v% LM shows Newtonian behavior with viscosity 14 mPa·s as the slope is very close to the unity in the logarithmic graph. When the LM volume fraction is increased to 30%, the viscosity at low shear rate increase more than 10 times. At 50 v/v%, the yield stress and shear thinning behavior appear to be stronger as typical characteristics of non-Newtonian fluids. For delicate printing of PaLMP at high resolution, 10 v/v% PaLMP with low viscosity is used throughout the demonstration.

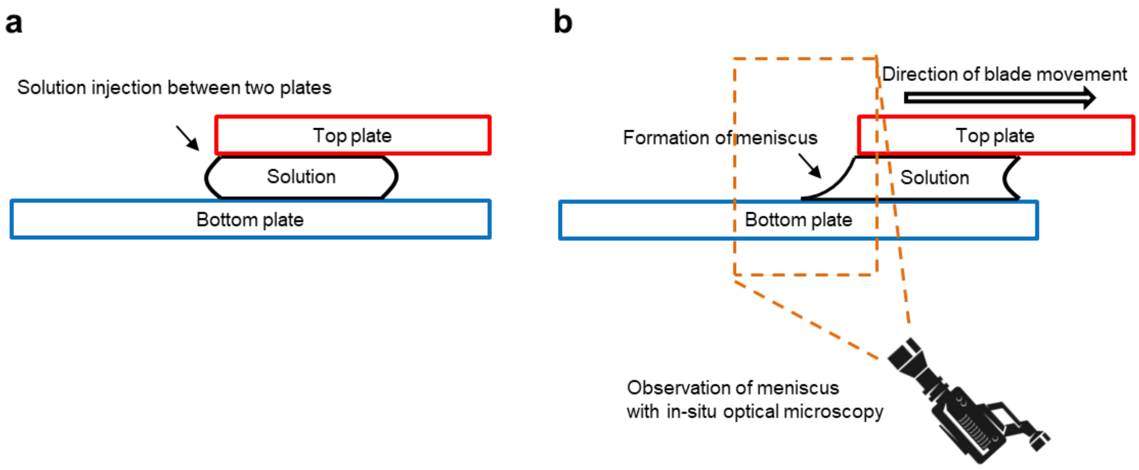

Supplementary Fig. 9| Schematic illustration meniscus observation set-up. Solution was injected between two plates. **a**, The meniscus was formed in the solution when one of the plates moved laterally. **b**, In-situ microscopy was set up to observe the shape of the meniscus in real time.

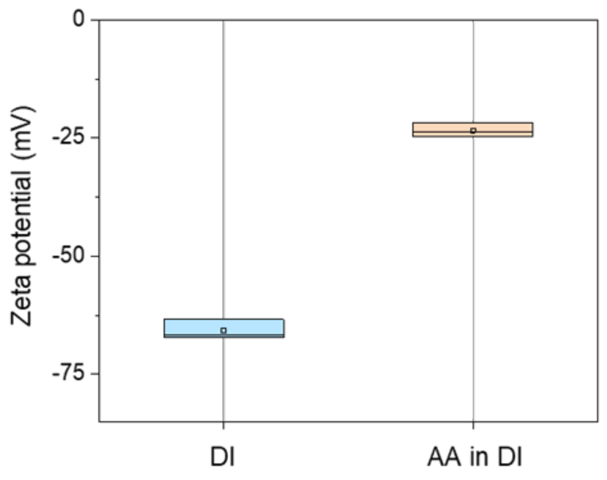

Supplementary Fig. 10| Zeta potential of PaLMP in different solvents.

Error bars indicate maximum and minimum values**.**

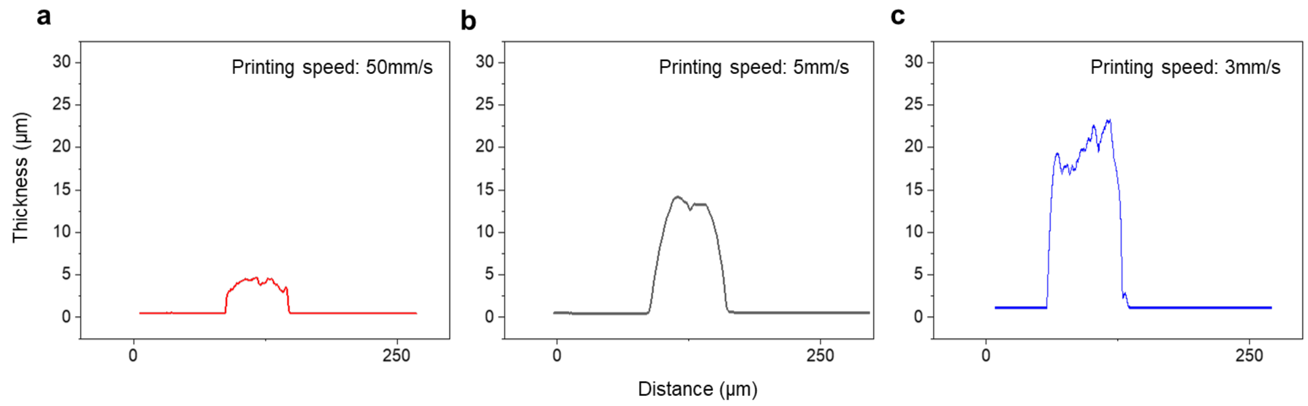

Supplementary Fig. 11| Surface profiled data of printed PaLMP with different thickness. **a-c**, Cross-sectional profile of printed PaLMP lines at different printing speeds.

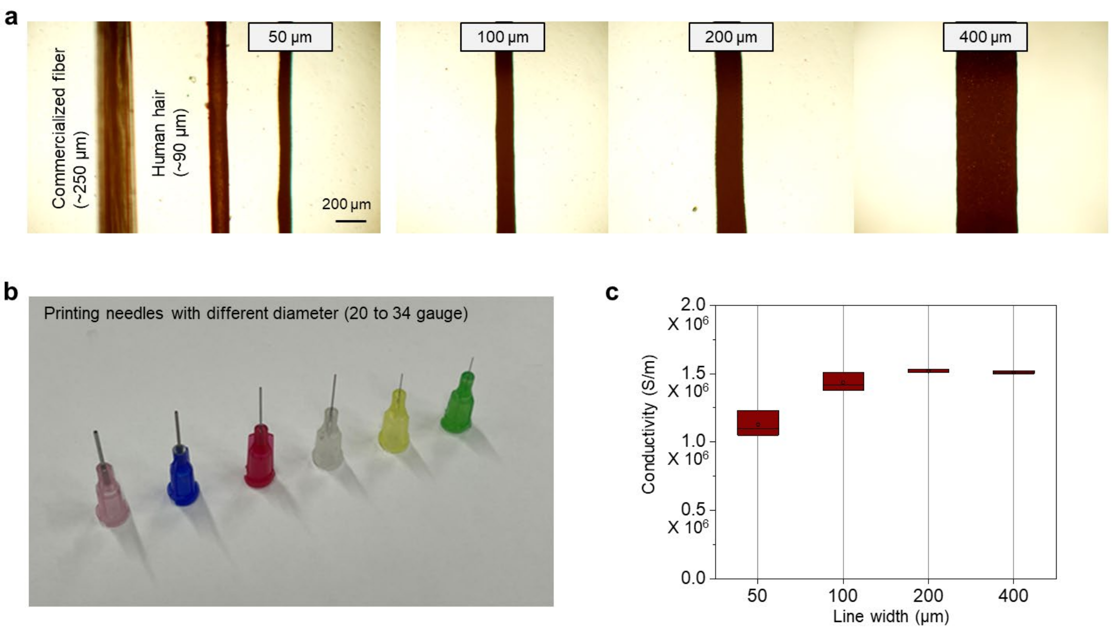

Supplementary Fig. 12| Printed PaLMP with different line width. **a**, OM image of printed PaLMP lines with different line width. **b**, Photograph of printing needles with different diameter. **c**, Conductivity of printed PaLMP line with different line width. Error bars indicate maximum and minimum values.

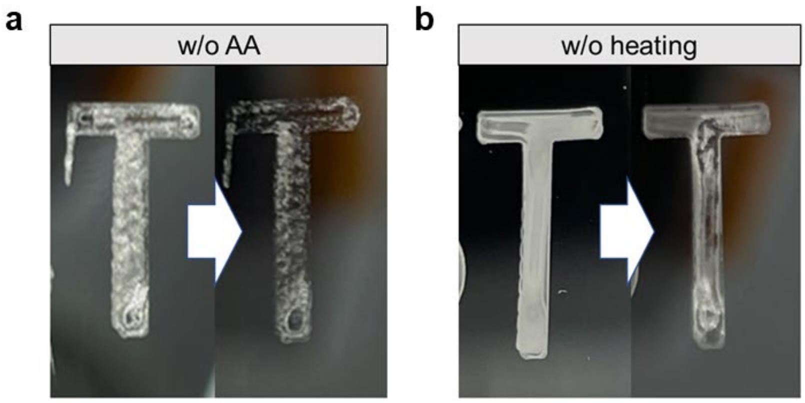

Supplementary Fig. 13| Peel-off test of films with different printing conditions. a, Printed PaLMP pattern with ink without AA. **b**, Printed PaLMP pattern without heating the substrate.

The left and right sides of the photograph show the PaLMP pattern before and after the peel-off test, respectively.

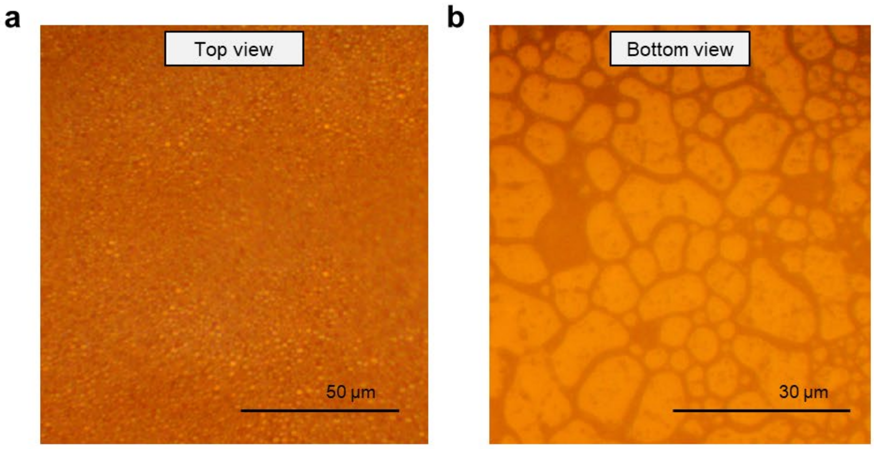

Supplementary Fig. 14| Top and bottom OM image of printed PaLMP. a, Top view. **b**, Bottom view.

The top layer of PaLMP film maintains its particle-packed morphology and the bottom layer of PaLMP film shows the sintered grain.

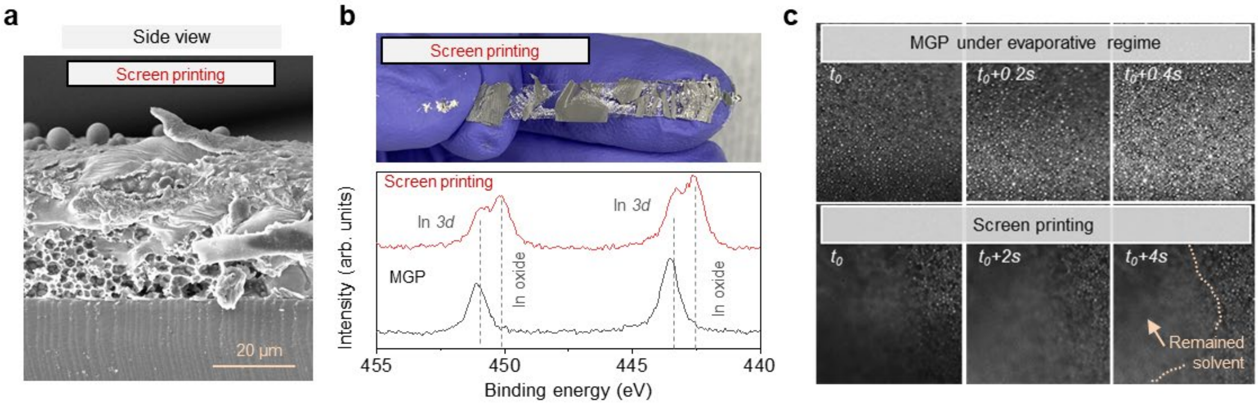

Supplementary Fig. 15| PaLMP film coated with screen printing. a, Side view SEM image of PaLMP deposited with screen printing. **b**, Photograph image of stretched PaLMP deposited with screen printing (top) and XPS spectra of deposited PaLMP with screen printing and MGP. **c**, In-situ OM observation of PaLMP deposition with MGP and screen printing.

For comparison with the meniscus-guided printing (MGP) process, screen printing was utilized to generate the PaLMP film (substrate: polydimethylsiloxane, PDMS). For screen printing, a mask was placed on top of the heated substrate at 70 °C, and liquid layer was formed in the opened areas by blade coating. Subsequent natural drying of the solvent generated the film. This film did not have the closely-packed particle assembled architecture; rather evidence of ruptured particles and cavities within the film were observed (**Supplementary Fig. 15a**). This film exhibited cracking and delamination under strain, as seen in **Supplementary Fig. 15b**, top. XPS show indium oxide peaks for this film, which are not present in MGP-based films (**Supplementary Fig. 15b, bottom)** (XPS data for other elements are presented in **Supplementary Fig. 16**). The brittleness of indium oxide may be contributing to the brittleness of the film. Real time optical microscopy show that for MGP, at the drying front near the contact line, the solution dries in 0.4 s. For screen printing, solvent took over 4 s to dry (**Supplementary Fig. 15c**). We project that the lengthened drying time for screen printing exposes the PaLMPs to excessive chemical annealing that reduces the gallium oxide shell, and oxidizes the indium.

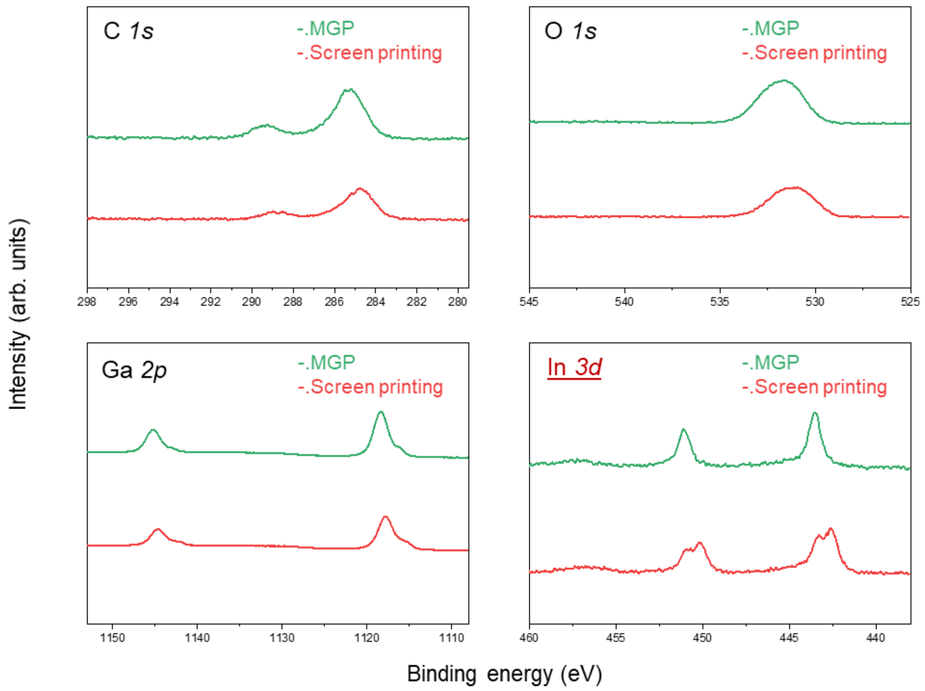

Supplementary Fig. 16| XPS of each element according to the printing method.

A notable change in XPS data was observed in the indium element.

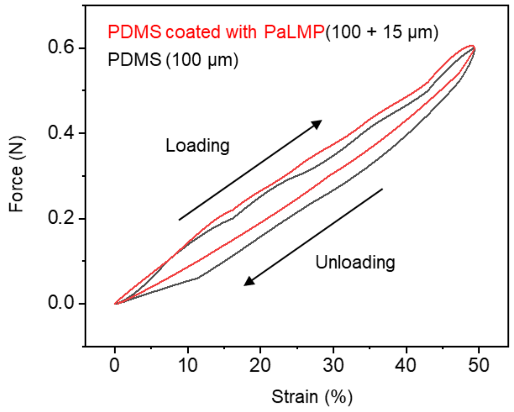

Supplementary Fig. 17| Force as a function of the strain of PDMS coated with PaLMP.

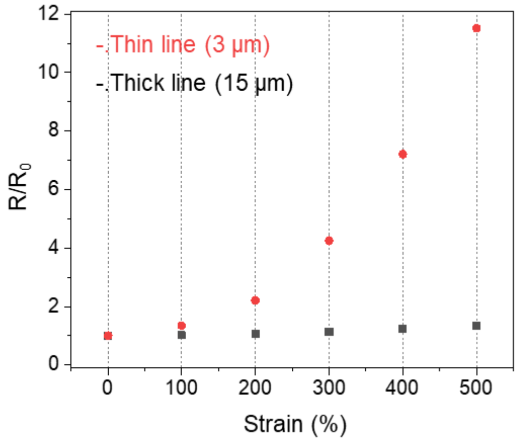

Supplementary Fig. 18| Normalized resistance according to strain with thin and thick printed PaLMP line.

Printed line dimensions: 0.3 mm X 20 mm

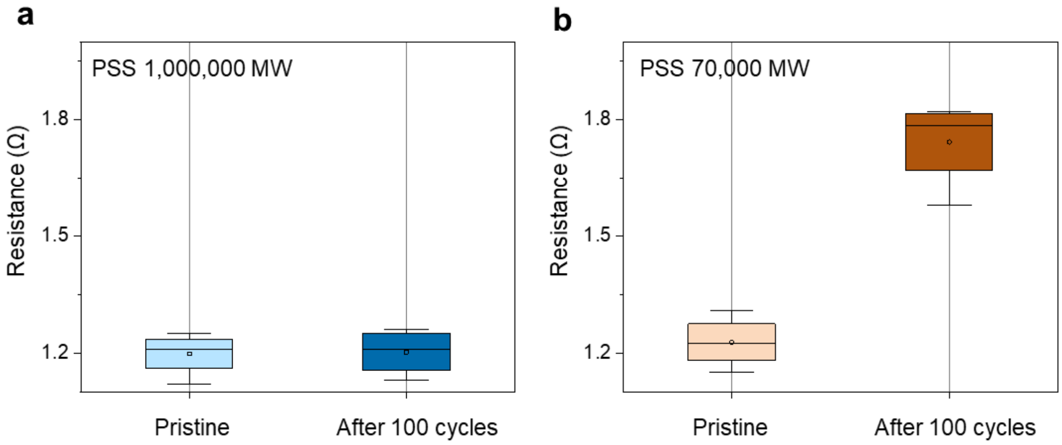

Supplementary Fig. 19| Resistance change after strain cycles according to molecular weight (MW) of PSS. a, MW of 1,000,000. **b**, MW of 70,000.

Printed line size: 0.3 mm X 20 mm

Error bars indicate maximum and minimum values**.**

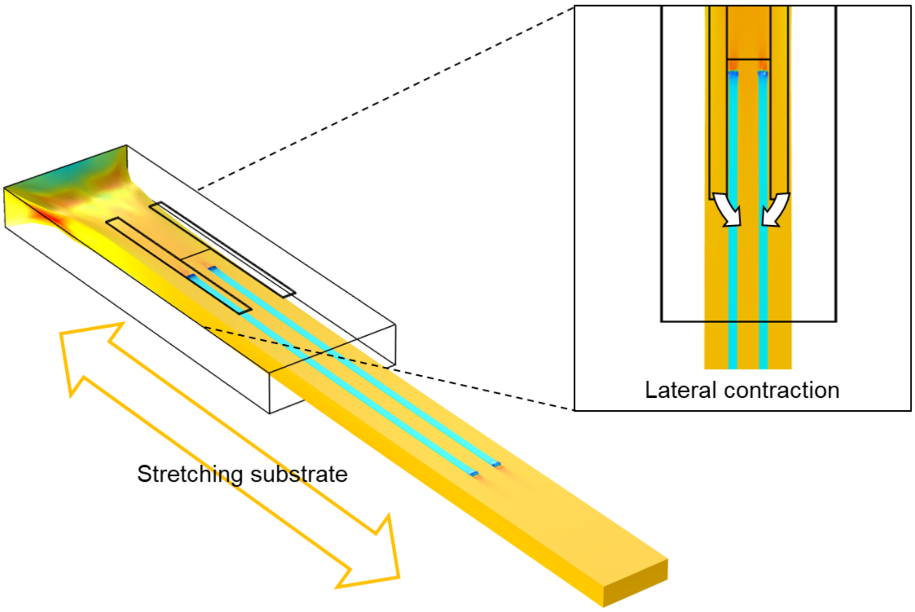

Supplementary Fig. 20| Simulation of Poisson effect on printed line.

When the substrate is stretched, the distance between the printed lines is diminished according to Poisson's ratio as shown in the COMSOL simulation. When the distance between the lines is 50 µm, the application of 200% strain on the substrate may cause merging between the liquid metal lines.

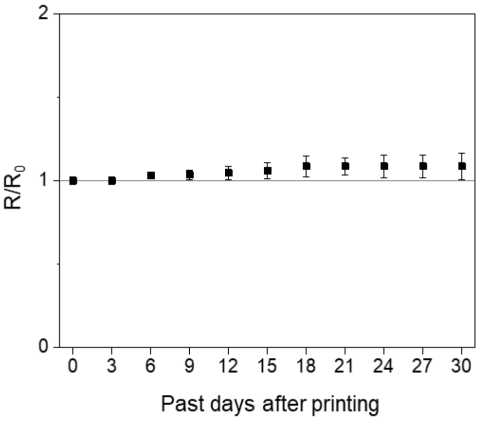

Supplementary Fig. 21| Resistance variation of printed PaLMP line for 30 days.

Error bars indicate maximum and minimum values**.**

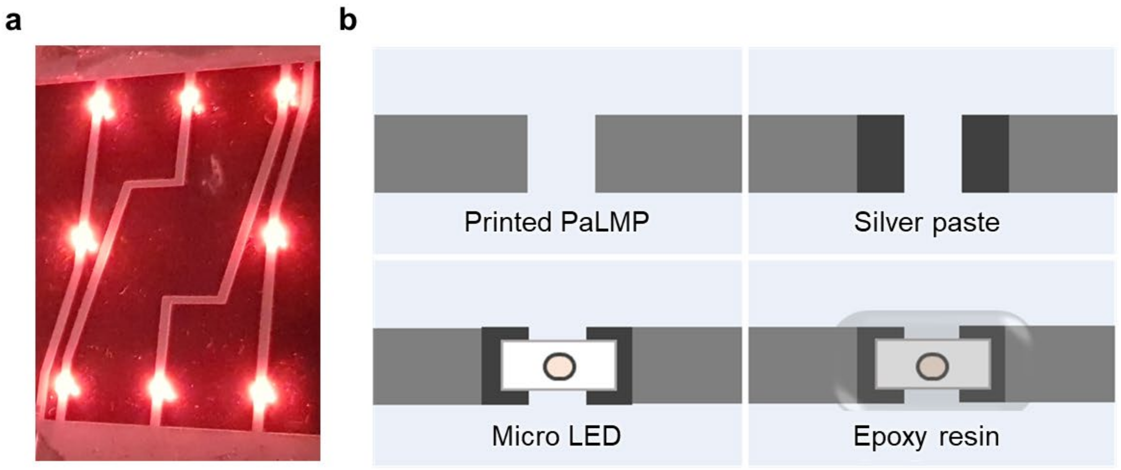

Supplementary Fig. 22| Integration of commercialized micro LED into PaLMP. **a**, Photograph of micro LED integrated on the PaLMP-based interconnect. **b**, Schematic illustration of micro LED integration with PaLMP-based interconnect.

Unlike bulk LM, stabilized PaLMPs can be easily integrated with conventional electronics with conductive adhesive such as silver paste**.**

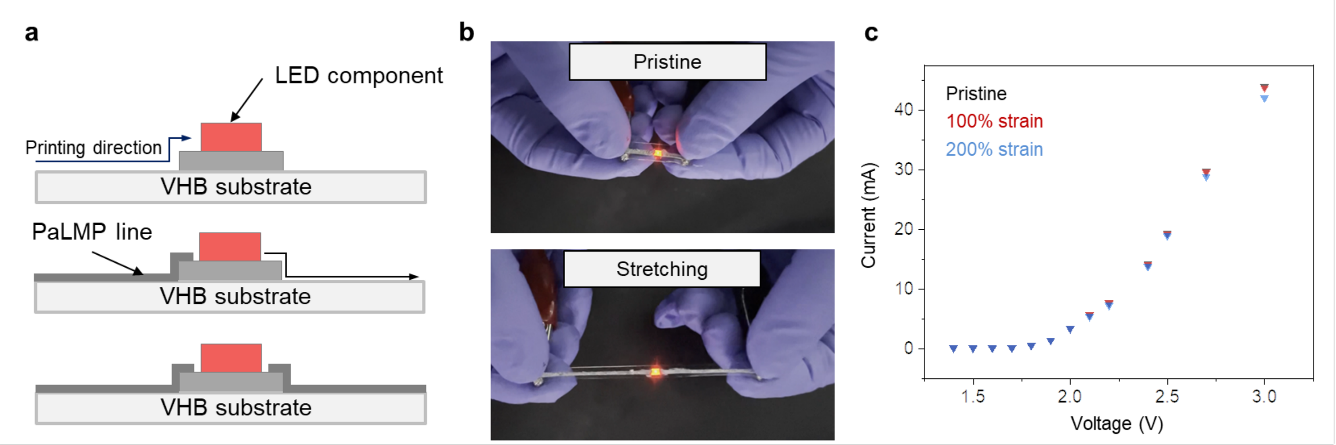

Supplementary Fig. 23| Conventional LED integrated with PaLMP without conductive paste. **a**, Schematic illustration of the integration of PaLMP and LED. **b**, Photograph of PaLMP before and after application of strain. **c**, I-V curve of the LED connected with PaLMP interconnect according to strain.

Conventional electronics components can be electrically connected without conductive epoxy or paste and shows stable operation under the application of strain.

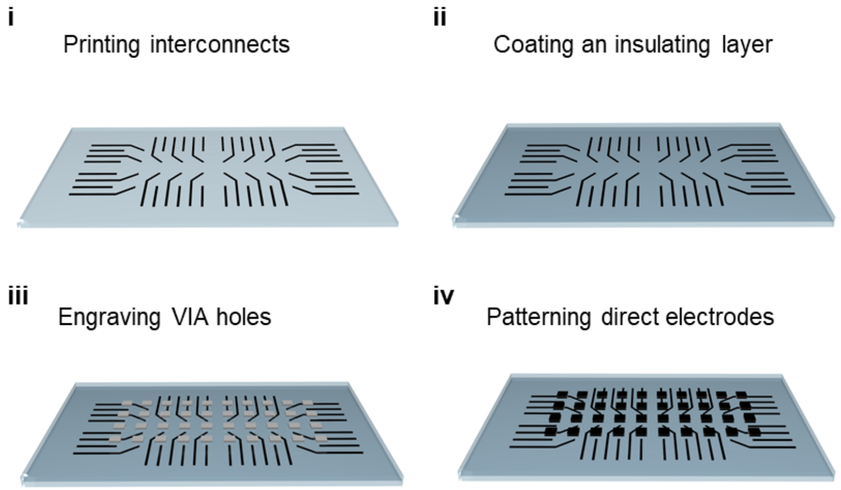

Supplementary Fig. 24| Schematic illustration of the overall fabrication process of PaLMP-based multilayer pressure sensing array.

The detailed fabrication method is presented in the method section.

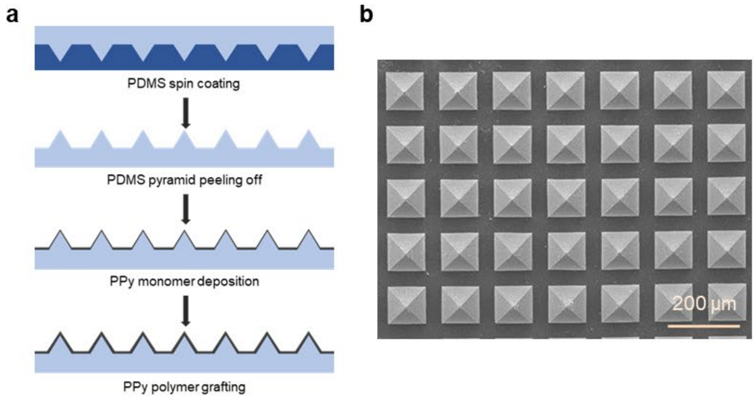

Supplementary Fig. 25| Fabrication process of pyramid-structured pressure sensor coated with conductive polymer. **a,** Schematic illustration of the overall fabrication process. **b**, SEM image of Pyramid-structured pressure sensor.

The detailed fabrication method is presented in the method section.

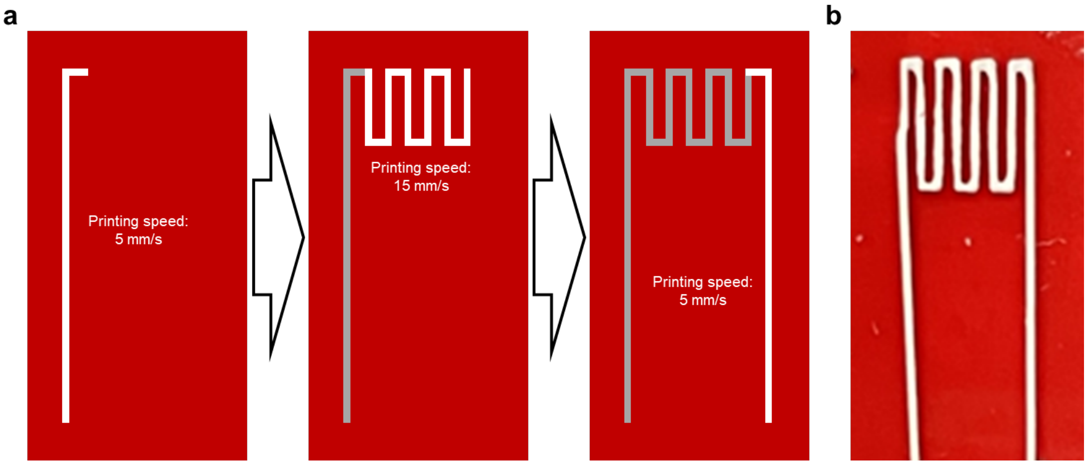

Supplementary Fig. 26| PaLMP-based artificial finger. a, Schematic illustration of the overall fabrication process. **b**, Photograph of the artificial finger with a serpentine resistor and interconnects.

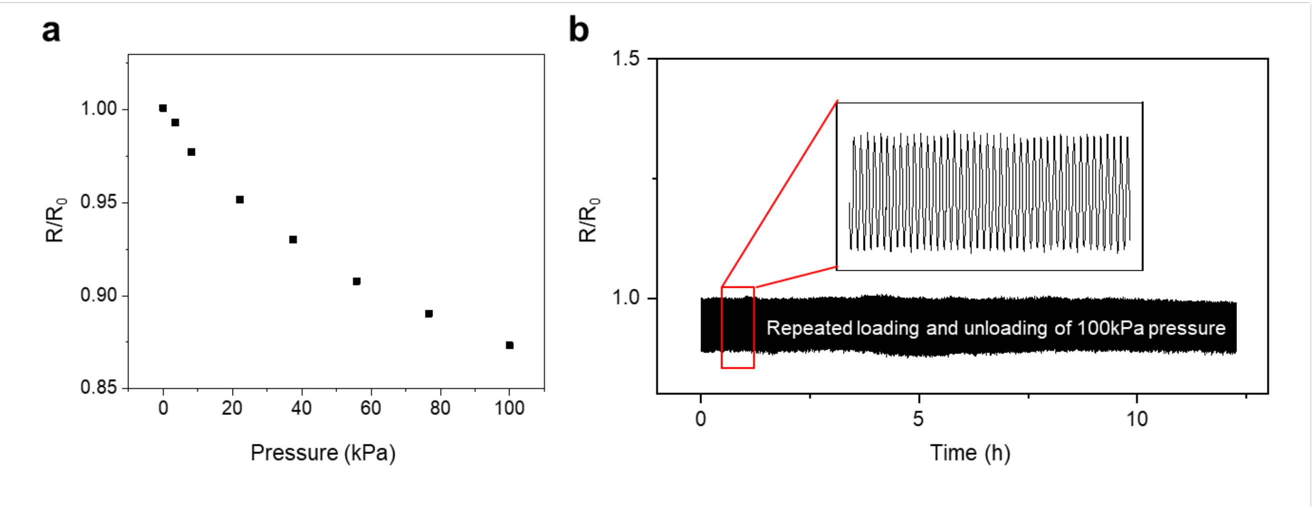

Supplementary Fig. 27| Pyramid-structured pressure sensor characterization. a, Normalized resistance according to pressure. **b**, Normalized resistance variation of pressure sensor laminated on PaLMP-based direct electrode under repeated application of 100 kPa.

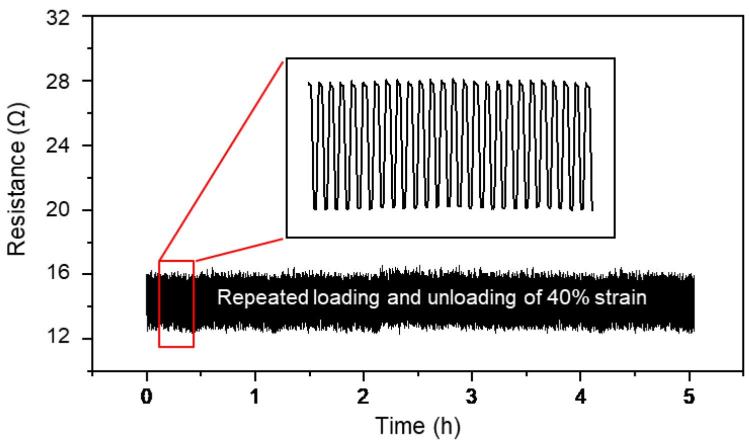

Supplementary Fig. 28| Resistance variation of PaLMP-based artificial finger under repeated application of 40% strain.

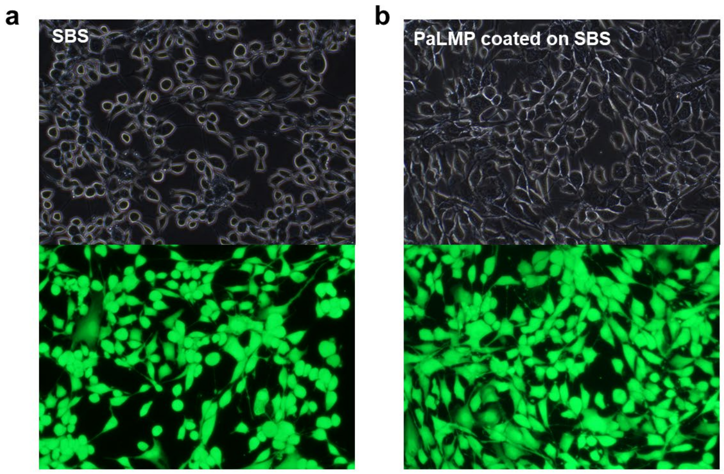

Supplementary Fig. 29| Biocompatibility of PaLMP film. a, Bright field and fluorescent images of 3T3 cells on SBS film **b**, Bright field and fluorescent images of 3T3 cells on PaLMP coated SBS film.

The proliferation of 3T3 cells on PaLMP indicates its biocompatibility suitable for wearable application.

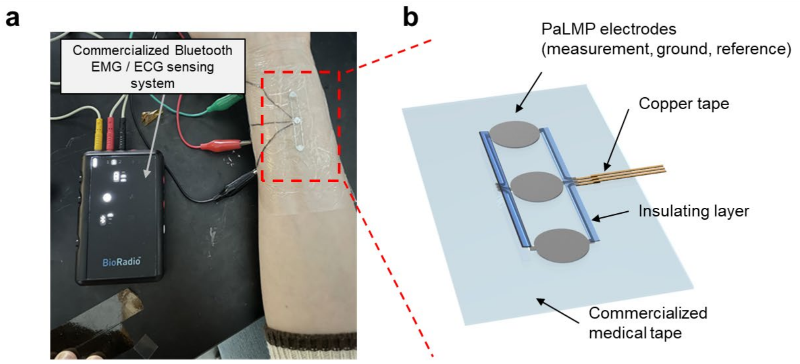

Supplementary Fig. 30| Experimental setup for EMG measurement. a, Photograph of setup for monitoring EMG. **b**, Schematic illustration of PaLMP-based EMG sensor.

A commercialized Bluetooth sensing device (BioRadio, Great Lakes NeuroTech.) was used for acquiring physiological signals (EMG, ECG).

Supplementary Note

Clogging simulation

Under pressure driven flow, the liquid/liquid interface departs from its spherical shape and the surface tension disturbs the neighboring flow. The deformation is much easier for bigger drops and the size of LMP drop is growing by its low dispersion stability. Here, we calculated the 'printing pressure' varying the size of single drop using the finite element method with COMSOL Multiphysics (COMSOL Inc., v5.6). Because the bottle-neck geometry between nozzle body and nozzle tip is well known as the region showing clogging[10-12], we focused on the axi-symmetric 2D bottle neck geometry for the calculation efficiency (**Supplementary Fig. 6**).

Governing equations

Navier-stokes equations are used to describe incompressible Newtonian fluids in geometry shown in **Supplementary Fig. 6**. Because $Re = 2\rho UR/\mu = 0.3$ ($R = 50\ \mu$m and $U = 6$ mm/s), the turbulent effect is not included[13]. For all liquids, time (t) dependent momentum and mass conservation equations are solved as

$$\rho\left(\frac{\partial \boldsymbol{u}}{\partial t} + \boldsymbol{u}\cdot\nabla\boldsymbol{u}\right) = -\nabla p + \nabla\cdot\mu(\nabla\boldsymbol{u} + \nabla\boldsymbol{u}^T) + \boldsymbol{F}_{st} \qquad (1)$$

$$\nabla\cdot\boldsymbol{u} = 0 \qquad (2)$$

where $\boldsymbol{u}$ is the flow velocity; p is the pressure. Liquid properties follow **Supplementary Table 2**. The equation (1) is coupled with multiphase model by $\boldsymbol{F}_{st}$, the surface tension force.

$$\boldsymbol{F}_{st} = \nabla\cdot[\gamma\{\boldsymbol{I} + (-\boldsymbol{n}\boldsymbol{n}^T)\}\delta] \qquad (3)$$

where γ is the interfacial tension between aqueous acetic acid and EGaIn; $\boldsymbol{I}$ is the identical tensor; $\boldsymbol{n}$ is the normal vector; δ is the Kronecker delta. $\boldsymbol{n}$ is determined by the shape of interface while its calculation is described in the next paragraph. Even if the oxide layer is known to reduce interfacial tension with water, it is negligible in acetic acid (pH<3)[14].

Level-set model is used to describe the transport of the interface between immiscible fluids[15-17]. In this model, the parameter ϕ is a function presenting the phase is dispersed (liquid metal) or continuous (solvent)

phase. ϕ is zero at the continuous phase and unity at the dispersed phase. The flux of phase variable can is solved as

$$\frac{\partial \phi}{\partial t} + \boldsymbol{u} \cdot \boldsymbol{\nabla}\phi = \lambda \boldsymbol{\nabla} \cdot \{\varepsilon \boldsymbol{\nabla}\phi + \phi(1-\phi)\boldsymbol{n}\} \tag{4}$$

where $\boldsymbol{n} = \frac{\nabla \phi}{|\nabla \phi|}$. ε is the thickness of interface and is calculated from the mesh size as $h_c/2$, where h_c is the characteristic mesh size in the interface. λ is the reinitialization factor and is determined as the typical flow speed (6 mm/s). One can find the advection of the interface as the second term of LHS, which means that equation (4) is also coupled with equation (1) and (2). In the RHS, the first term is the numerical diffusion of the interface and the second term is compressive flux[15, 16, 18]. For the smooth transition in liquid properties across the interface, the viscosity and density are calculated from ϕ as

$$\rho = \rho_1 + (\rho_2 - \rho_1)\phi \text{ and } \mu = \mu_1 + (\mu_2 - \mu_1)\phi \tag{5}$$

where 1 and 2 denote for continuous and dispersed phase, respectively. The interface is moving at the nozzle wall while the bulk flow is no-slip. To give such mobility to the interface, the Navier slip stress (τ_{slip}) is given to the wall as

$$\tau_{slip} = \frac{\mu}{l_{slip}} u_{slip} \tag{6}$$

where u_{slip} is the slip velocity and calculated with the normal vector and velocity field as $\boldsymbol{u} \cdot (\boldsymbol{u} \cdot \boldsymbol{n})\boldsymbol{n}$. l_{slip} is the slip length and used the size of h_c. Furthermore, to keep the wettability between the liquid metal drop and the nozzle wall, the final expression at the wall boundary can be written as

$$\boldsymbol{F}_{st} = \gamma\delta(\boldsymbol{n}_{\mathrm{w}} \cdot \boldsymbol{n} - \cos\theta_0)\boldsymbol{n} - \boldsymbol{\tau}_{slip} \tag{7}$$

where θ_0 is the contact angle of liquid metal on the nozzle inner surface and is fixed as perfectly non-wetting condition (θ_0=180°). $\boldsymbol{n}_{\mathrm{w}}$ is the normal vector of the wall boundary wall. $\boldsymbol{\tau}_{slip}$ in equation (6) is vector form in equation (7) because the slip velocity is calculated in each boundary position for each element of interface. Finally equation (1) ~ (7) are simultaneously calculated in the time ranging from 0 to 10 ms with interval 0.01 ms.

The entire meshing is done with the 'finer' option of the built-in tool of COMSOL, resulting in 65,058

elements. The mesh size is tested in two steps. First, we tested the convergence with fluidics without liquid/liquid interface and second tested with bubble rising problem[19]. In the **Supplementary Fig. 7**, the velocity profile converges to finer mesh condition, and the bubble shape excellently agrees with the experimental result. For the bubble rising simulation, liquid properties and geometry follow the experimental conditions.

Printing pressure for different drop sizes

Changing the radius of liquid metal droplet, the printing pressure is calculated as **Fig. 2D**. The printing pressure is calculated from the pressure difference between inlet and outlet of model nozzle. The simulation results separated into two groups; the drop totally blocks the nozzle tip (red group) or does not (black group). The maximum printing pressure is displayed for the printing pressure for the red group; whereas the final pressure is displayed for the black group. This is because the black group undergo stress overshoot right before the drop deformation that originated from the surface tension of EGaIn. The local pressure gradient within the simulation geometry (**Supplementary Fig. 6**) is suggested as the working range of printing pressure.

Supplementary References

[1] Park, Y. G., An, H. S., Kim, J. Y. & Park, J. U. High-resolution, reconfigurable printing of liquid metals with three-dimensional structures. *Sci. Adv.* 5, (2019).

[2] Ladd, C., So, J. H., Muth, J. & Dickey, M. D. 3D Printing of Free Standing Liquid Metal Microstructures. *Adv. Mater.* **25**, 5081-5085, (2013).

[3] Boley, J. W., White, E. L., Chiu, G. T. C. & Kramer, R. K. Direct Writing of Gallium-Indium Alloy for Stretchable Electronics. *Adv. Funct. Mater.* **24**, 3501-3507, (2014).

[4] Veerapandian, S. *et al.* Hydrogen-doped viscoplastic liquid metal microparticles for stretchable printed metal lines. *Nat. Mater.* **20**, 533-+, (2021).

[5] Ford, M. J. *et al.* Controlled Assembly of Liquid Metal Inclusions as a General Approach for Multifunctional Composites. *Adv. Mater.* **32**, (2020).

[6] Tang, L. X., Mou, L., Zhang, W. & Jiang, X. Y. Large-Scale Fabrication of Highly Elastic Conductors on a Broad Range of Surfaces. *Acs Appl. Mater. Interfaces* **11**, 7138-7147, (2019).

[7] Xu, J. Y. *et al.* Printable and Recyclable Conductive Ink Based on a Liquid Metal with Excellent Surface Wettability for Flexible Electronics. *Acs Appl. Mater. Interfaces* **13**, 7443-7452, (2021).

[8] Zhou, L. Y. *et al.* All-Printed Flexible and Stretchable Electronics with Pressing or Freezing Activatable Liquid-Metal-Silicone Inks. *Adv. Funct. Mater.* **30**, (2020).

[9] Li, Y. Y. *et al.* Printable Liquid Metal Microparticle Ink for Ultrastretchable Electronics. *ACS Appl. Mater. Interfaces* **12**, 50852-50859, (2020).

[10] Dressaire, E, & Alban Sauret. "Clogging of microfluidic systems." Soft Matter 13.1 (2017): 37-48.

[11] Zuriguel, Iker, et al. "Clogging transition of many-particle systems flowing through bottlenecks." *Sci. Rep.* **4**, 1-8, (2014).

[12] Trofa, M. *et al*. "Numerical simulation of clogging in a microchannel with planar contraction." *Physics of Fluids* **33**, 083320, (2021).

[13] Den Toonder, J. *et al.* "Reynolds number effects in a turbulent pipe flow for low to moderate Re." *Physics of Fluids* **9**, 3398-3409, (1997).

[14] Xu, Qin, *et al.* "Effect of oxidation on the mechanical properties of liquid gallium and eutectic gallium-indium." *Physics of fluids* **24**, 063101, (2012).

[15] Olsson, E. *et al.* "A conservative level set method for two phase flow." *Journal of computational physics* **210**, 225-246, (2005).

[16] Amani, A. *et al.* "Numerical study of droplet deformation in shear flow using a conservative level-set method." *Chemical Engineering Science* **207**, 153-171, (2019).

[17] Yiotis, A. *et al.* "Pore-scale effects during the transition from capillary-to viscosity-dominated flow dynamics within microfluidic porous-like domains." *Sci. Rep.* **11**, 1-16, (2021).

[18] González, Be. *et al* "Dynamic viscosities, densities, and speed of sound and derived properties of the binary systems acetic acid with water, methanol, ethanol, ethyl acetate and methyl acetate at T=(293.15, 298.15, and 303.15) K at atmospheric pressure." *Journal of Chemical & Engineering Data* **49**, 1590-1596, (2004).

[19] Liu, L. *et al.* "Experimental studies on the shape and motion of air bubbles in viscous liquids." *Experimental Thermal and Fluid Science* **62**, 109-121, (2015).
