# Supplementary material for: Rapid meniscus-guided printing of stable semi-solid-state liquid metal microgranular-particle for soft electronics
Source: Nat Commun. 2022 May 12;13:2643. doi: 10.1038/s41467-022-30427-z (PMC9098628; doi:10.1038/s41467-022-30427-z)
Supplement: Supplementary file 3 — Description of Additional Supplementary Files [file 41467_2022_30427_MOESM3_ESM.pdf]

File name: Supplementary Movie 1

Description: Printing of bare liquid metal particle-based ink

File name: Supplementary Movie 2

Description: Printing of polyelectrolyte-attached liquid metal particle-based ink
